# The cardiometabolic benefits of okra-based treatment in prediabetes and diabetes: a systematic review and meta-analysis of randomized controlled trials

**DOI:** 10.3389/fnut.2024.1454286

**Published:** 2024-12-12

**Authors:** Hossein Bahari, Mostafa Shahraki Jazinaki, Iman Rahnama, Ladan Aghakhani, Mohammad Reza Amini, Mahsa Malekahmadi

**Affiliations:** ^1^Transplant Research Center, Clinical Research Institute, Mashhad University of Medical Sciences, Mashhad, Iran; ^2^Student Research Committee, Mashhad University of Medical Sciences, Mashhad, Iran; ^3^Binaloud Institute of Higher Education, Mashhad, Iran; ^4^Laparoscopy Research Center, Shiraz University of Medical Sciences, Shiraz, Iran; ^5^Student Research Committee, Department of Clinical Nutrition and Dietetics, Faculty of Nutrition Sciences and Food Technology, National Nutrition and Food Technology Research Institute, Shahid Beheshti University of Medical Sciences, Tehran, Iran; ^6^Nutrition and Food Security Research Center, Isfahan University of Medical Sciences, Isfahan, Iran; ^7^Imam Khomeini Hospital Complex, Tehran University of Medical Sciences, Tehran, Iran

**Keywords:** *Abelmoschus esculentus*, okra, diabetes, cardiometabolic risk factors, meta-analysis

## Abstract

**Background:**

This systematic review and meta-analysis examine the effects of okra consumption on cardiometabolic risk factors in individuals with prediabetes and diabetes. Okra is a widely consumed vegetable with potential health benefits, and understanding its impact on metabolic parameters in these populations is important.

**Methods:**

A comprehensive search of the literature was conducted up to May 2024 in PubMed/Medline, Scopus, and Web of Science to find relevant randomized clinical trials (RCTs) by using following keyword: (“okra” OR “okras” OR “*abelmoschus esculentus*”) AND (“intervention” OR “controlled trial” OR “randomized” OR “randomized” OR “randomly” OR “clinical trial” OR “trial” OR “randomized controlled trial” OR “randomized clinical trial” OR “RCT” OR “blinded” OR “placebo” OR “Cross-Over” OR “parallel”). The selected trials were subjected to heterogeneity tests using the I^2^ statistic. Random effects models were examined based on the heterogeneity tests, and the pooled data were calculated as weighted mean differences (WMD) with a 95% confidence interval (CI). In this meta-analysis, all the analyses were performed by using the STATA version 17 software.

**Results:**

Of the 1,339 papers, nine eligible RCTs were included in the present meta-analysis. Our findings indicated that okra consumption significantly reduced total cholesterol (TC) levels (WMD: −14.40 mg/dL; (95% CI: −20.94 to −7.86); *p* < 0.001), low-density lipoprotein (LDL) (WMD: −7.90 mg/dL; (95% CI: −13.30 to −2.48); *p* = 0.004), fasting blood glucose (FBG) (WMD: −39.58 mg/dL; (95% CI: −61.60 to −17.56); *p* < 0.001), and hemoglobin A1C (HbA1c) (WMD: −0.46 mg/dL; (95% CI: −0.79 to −0.13); *p* = 0.005). Overall effect size showed that okra intake failed to change triglycerides (TG), high-density lipoprotein (HDL), Insulin, homeostatic model assessment for insulin resistance (HOMA-IR), systolic blood pressure (SBP), diastolic blood pressure (DBP), body weight, and body Mass Index (BMI) significantly.

**Conclusion:**

Okra decreased TC, LDL, FBG, and HbA1c levels in the intervention compared to the control group. A dose ≤3,000 mg/day caused a significant decrease in TG, TC, LDL, HbA1c, and a significant increase in HDL. More study is needed to determine the optimum dose and duration of intervention.

## 1 Introduction

Cardiometabolic disease (CMD) is a broad term that encompasses various chronic conditions that often occur together, including diabetes mellitus, cardiovascular disease (CVD), chronic kidney disease (CKD), High blood pressure, and stroke (1). These complex disorders are affected by a variety of factors, such as alterations in living conditions, dietary habits, and lifestyle choices, along with genetic and epigenetic influences that play a role in their development and progression (2).

CMDs are a major contributor to illness and mortality worldwide, imposing a substantial burden on individuals, healthcare systems, and society (1, 3). Over 60% of deaths from chronic kidney disease, diabetes, and cardiovascular disorders can be attributed to the presence of cardiometabolic risk factors like hypertension, inflammation, dyslipidemia, and obesity (3).

Nevertheless, cardiometabolic disorders can mostly be prevented, and it will be crucial to have a greater understanding of the factors involved in their development and to implement interventions to address these factors in order to combat the current epidemic. Therefore, numerous researchers have concentrated on unraveling non-pharmacological methods for managing cardiometabolic risk factors (3, 4).

Diet plays a crucial role in the development, prevention, and management of cardiometabolic disorders (4). In this regard, certain components found in plant-based foods are believed to offer significant advantages for cardiometabolic health (5). Okra (*Abelmoschus esculentus* L.), a member of the Malvaceae family, is a yearly shrub grown primarily in tropical and subtropical areas worldwide and is a popular crop for both gardens as well as farms (6). Based on the nutritional information, 100 grams of raw okra contains 33 kilocalories, 7.45 g of carbohydrates, 1.93 g of protein, 3.2 g of dietary fiber, 299 mg of potassium,0.027 g of polyunsaturated fat, and 0.026 g of unsaturated fat (7). In recent times, okra has been utilized not just for its nutritional benefits, but also for its nutraceutical and therapeutic properties, thanks to the presence of several crucial bioactive compounds and their corresponding bioactivities (6).

The leaves, buds, flowers, pods, stems, and seeds of okra are valued for their various medicinal uses in both traditional and modern medicine, making it a valuable crop (8). Previous studies have demonstrated that okra possesses a diverse range of pharmacological effects, such as anti-inflammatory, antioxidant, gastroprotective, immunomodulatory, neuroprotective, antibacterial, lipid-lowering, anticancer, and antidiabetic properties (8, 9).

A systematic review, which included 26 papers including animal and *in vitro* studies, suggested that okra has the potential to reduce key inflammatory mediators such as C-reactive protein (CRP), interleukin-1β (IL-1β), interleukin-6 (IL-6), and tumor necrosis factor-alpha (TNF-*α*) (10).

The antihyperglycemic effect of okra has been extensively researched by numerous researchers in recent years (11–13). A recent systematic review and meta-analysis of eight studies involving 331 patients concluded that okra treatment is beneficial for glycaemic control in pre-diabetic and T2D patients, as evidenced by a significant decrease in fasting blood glucose levels (FBG) (14).

However, the effects of consuming okra on cardiometabolic factors are still not fully understood. In order to fill this knowledge gap, we performed a thorough systematic review and meta-analysis of published randomized controlled trials (RCTs) to assess the impact of consuming okra products on cardiovascular factors.

## 2 Materials and methods

All steps that were done for conducting this systematic review were designed and performed according to the Preferred Reporting Items of Systematic Reviews and Meta-Analysis (PRISMA) framework (15). Also, the protocol for conducting this review was registered in the PROSPERO database with registration ID: CRD42024547242.

### 2.1 Search strategy and study selection

A comprehensive systematic search was performed in the databases including PubMed, Scopus, and Web of Science (ISI) to find eligible studies up to May 2024. This search was restricted to English language papers while having no time restriction. This systematic search was designed using the following keywords (including MeSH and Non-MeSH terms): (“okra” OR “okras” OR “*abelmoschus esculentus*”) AND (“intervention” OR “controlled trial” OR “randomized” OR “randomized” OR “randomly” OR “clinical trial” OR “trial” OR “randomized controlled trial” OR “randomized clinical trial” OR “RCT” OR “blinded” OR “placebo” OR “Cross-Over” OR “parallel”).

To avoid missing eligible trials, the reference lists of related articles were completely reviewed, and also google Scholar search engine was manually searched. The studies obtained by the initial search were screened by two researchers (H.B and M.Sh.J), independently.

### 2.2 Eligibility criteria

The eligibility criteria of this review were designed based on the PICOS framework (Participant: adults; Intervention: Okra intake; Comparison: control group; Outcome: lipid profile, glycemic control, blood pressure, and obesity indices; Study: randomized controlled trials (RCTs)).

Studies were included in this review if they met the following criteria:

- Studies with RCT design (parallel or cross-over) that investigate the impact of Okra (*Abelmoschus esculentus*) intake on lipid profile, glycemic control markers, blood pressure, or obesity indices
- Studies with at least 2 weeks of intervention
- Studies conducted on the adult populations (≥18 years) with prediabetes or type 2 diabetes
- Having appropriate control groups (that had not any difference with the intervention group except the Okra intake).
- Outcome level changes were reported following the intervention as mean ± SD (or outcome levels were reported at the beginning and the end of the intervention)

Trials with the following characteristics were excluded from this review: (a) Trials conducted on non-adults (<18 years), (b) studies with multi-intervention, (c) animal studies, (d) or trials without RCT design including observational studies, review articles, short communications, and letters to the editors.

### 2.3 Data extraction

The following related data was extracted from included trials by two researchers independently (H.B and M.Sh.J): first author name, publication year, country, gender, population characteristics (health status, mean age, mean BMI), sample size (total and in each group), intervention features (type, dosage, and duration), type of control groups, and mean changes ± SD of outcome levels in both intervention and control group. Disagreement items were discussed until a consensus was reached.

### 2.4 Quality assessment

This risk of bias in included trials was assessed by two researchers independently (H.B and I.R), using the Cochrane Collaboration RoB2 tool (16). This tool contains 5 domains including, Bias arising from the randomization process, Bias in selection of the reported result, Bias due to deviations from intended interventions, Bias in measurement of the outcome, and Bias due to missing outcome data. The risk of bias overall and in each subclass was categorized into three levels: High, some concerns, and Low. Disagreements were resolved by consulting the third author (M.M).

### 2.5 Data synthesis and statistical analysis

In this meta-analysis, the overall effect sizes were estimated as weighted mean differences with a 95% confidence interval by using the weighted mean differences (WMD) and the SD of measures according to the DerSimonian and Laird method (17). In the cases where mean changes in outcomes were not reported, it was calculated by subtracting the outcome levels at the beginning of the study from measures at the end of the intervention (mean change = final values − baseline values) (18). Also, SDs were calculated by using the following formula: square root [(SDbaseline)^2^ + (SDfinal)^2^ − (2 × R × SDbaseline × SDfinal)] (19). A correlation coefficient of 0.9 was regarded as an R-value that ranged from 0 to 1 (19). Hozo et al. approach was applied to convert the 95% confidence intervals (95% CIs), standard errors (SEs), and interquartile ranges (IQRs) to SD (16, 20, 21). Heterogeneity among the pooled studies was evaluated by Cochran’s Q test. Also based on the calculated I-squared statistic heterogeneity was measured (22). In this review, *p*-value <0.05 or I^2^ > 40% was deemed as significant heterogeneity among the included trials (23). Subgroup analysis was performed to find the source of heterogeneity among the included trials for each outcome based on the following predefined criteria (24), Health Status (type 2 diabetes and prediabetes), baseline BMI (normal, overweight, and obesity), intervention duration (>8 and ≤ 8 weeks), intervention type (Okra fruit, Okra extract, and Okra powder), and Okra powder dosage (>3,000 vs. ≤3,000 mg/day). Statistical analysis of this meta-analysis was performed by using the STATA, version 17 (Stata Corp, College Station, TX). Also, in all of the analyses, *p*-values <0.05 were identified as statistically significant.

### 2.6 Certainty assessment

The certainty of findings was evaluated by applying the GRADE (Grading of Recommendations Assessment, Development, and Evaluation) protocol (25). This framework assessed the quality of the evidence in the 5 sections including, risk of bias, inconsistency, indirectness, Imprecision, and Publication Bias. The quality of the evidence in each section was identified as having no serious limitations, serious limitations, and very serious limitations. The overall certainty for each evidence was categorized into four levels: very low, low, moderate, and high.

## 3 Results

### 3.1 Study selection

**Figure 1:**
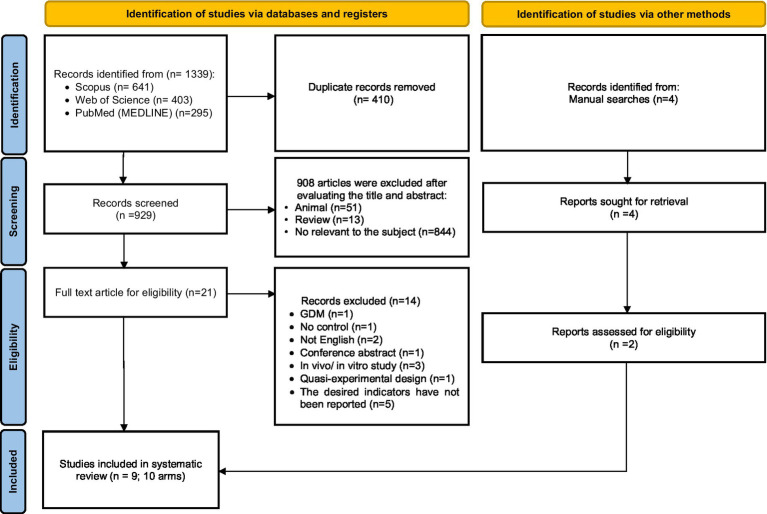
Flow chart of study selection for inclusion trials in the systematic review.

Among the 1,339 studies found by a comprehensive search in the databases, 410 duplicate studies were excluded. Then, 929 studies were screened based on their titles and abstracts. The full text of 21 studies was read to assess eligibility criteria. Fourteen studies due to being conducted on pregnant women with GDM (*n* = 1), not having a suitable control group (*n* = 1), Conference abstract (*n* = 1), non-English language studies (*n* = 2), *in vivo* or *in vitro* studies (*n* = 3), studies with quasi-experimental design (*n* = 3), and not reporting changes in related outcomes after Okra consumption (n = 3). Also, 4 studies were found by manual search, 2 of which met the inclusion criteria for this review. Finally, 9 studies (10 arm treatments) with 540 participants were included in this systematic review and meta-analysis (Figure 1) (12, 26–33).

### 3.2 Study characteristics

The studies included in this review were published between 2017 (26), and 2024 (31–33). Among the included studies, one study was conducted in the Philippines (26), one in Indonesia (27), and the rest in Iran (12, 28–33). In one study, the participants had prediabetes (31), and the rest had type 2 diabetes. The study sites varied from 20 (27) to 99 participants (12) and the duration of the intervention varied from 2 (27) to 12 weeks (30). Also, the mean age of participants receiving Okra in the included studies was between 45.8 ± 6 (31) and 62 ± 7 years (29, 32, 33) and their body mass index was between 24.9 ± 3 (28), and 30.3 ± 5 kg/m^2^ (29, 33). All included trials had a parallel design and were conducted on both sexes. Among the included studies, 8 blinding of the intervention subjects was done, and in only one study, blinding of the participants was not done (27). Furthermore, among the included treatment arms, dried Okra extract was used in 3 (29) (32, 33), boiled Okra in one (27), steamed Okra in one (27), and Okra powder was used in the rest to intervene on the participants (12, 26, 28, 30, 31). The dose of Okra powder used varied from 600 (26) to 4,000 mg/day (12). The characteristics of the included trials are summarized in Table 1.

**Table 1:** Characteristic of included studies in meta-analysis.

Studies	Country	Study design	Participant	Sex	Sample size		Trial duration (Week)	Means Age		Means BMI		Intervention		
					IG	CG		IG	CG	IG	CG	Type	Dose (mg/day)	Control group
Labadnoy et al. (26)	Philippines	Parallel, R, PC, DB	T2DM	M/F (M:8, F:16)	12	12	4	54	48.3	27.4	26.8	Okra powder	600	Placebo
Khodija et al. (27)	Indonesia	Parallel, R, CO, NR	T2DM	M/F (M:7, F:21)	12	8	2	45–65	45–65	NR	NR	Steamed okra	40,000	Nothing
Khodija et al. (27)	Indonesia	Parallel, R, CO, NR	T2DM	M/F (M:7, F:21)	12	8	2	45–65	45–65	NR	NR	Boiled okra	40,000	Nothing
Moradi et al. (28)	Iran	Parallel, R, PC, DB	T2DM	M/F (M:36, F:44)	25	23	8	54.26	53.33	24.9	25.6	Yogurt + okra powder	10,000	Yogurt
Saatchi et al. (12)	Iran	Parallel, R, PC, DB	T2DM	M/F (M:36, F:63)	50	49	8	57.7	58.3	30.2	31.1	Okra powder	4,000	Placebo
Nikpayam et al. (29)	Iran	Parallel, R, PC, TB	DN patients	M/F (M:16, F:39)	30	25	10	62	61.6	30.3	28.6	Dried okra extract powder	125	Placebo
Tavakolizadeh et al. (30)	Iran	Parallel, R, PC, DB	T2DM	M/F (M:30, F:64)	48	46	12	53.8	52.8	28.6	29.5	Okra powder	3,000	Placebo
Nikpayam et al. (33)	Iran	Parallel, R, PC, TB	DN patients	M/F (M:16, F:39)	30	25	10	62	61.6	30.3	28.6	Dried okra extract powder	125	Placebo
Bahreini et al. (32)	Iran	Parallel, R, PC, TB	DN patients	M/F (M:16, F:39)	30	25	10	62	61.6	30.3	28.6	Dried okra extract powder	125	Placebo
Afsharmanesh et al. (31)	Iran	Parallel, R, PC, DB	Pre-diabetic Adults	M/F (M:30, F:40)	35	35	8	45.8	45.6	NR	NR	Okra powder	3,000	Placebo

IG, intervention group; CG, control group; TB, triple-blinded; DB, double-blinded; PC, placebo-controlled; CO, controlled; R, randomized; T2DM, type 2 diabetes mellitus; DN, diabetic nephropathy; NR, not reported.

### 3.3 Quality assessment

Risk of Bias Assessment done by following the ROB 2 framework; reported some concerns for the overall risk of bias for two studies (26, 31), the overall high risk of bias for one study (27), and the overall risk of bias was low in the rest of the included studies. The details of the risk of bias assessment for each study in each subclass of the Rob 2 tool are summarized in Table 2.

**Table 2:** Risk of bias assessment.

Study	Bias arising from the randomization process	Bias in selection of the reported result	Bias due to deviations from intended interventions	Bias in measurement of the outcome	Bias due to missing outcome data	Overall risk of bias
Labadnoy et al. (26)	L	H	L	U	L	Some concerns
Khodija et al. (27)	L	H	U	U	L	High
Moradi et al. (28)	L	L	L	U	L	Low
Saatchi et al. (12)	L	L	L	U	L	Low
Nikpayam et al. (29)	L	L	L	L	L	Low
Tavakolizadeh et al. (30)	L	L	L	U	L	Low
Nikpayam et al. (33)	L	L	L	L	L	Low
Bahreini et al. (32)	L	L	L	L	L	Low
Afsharmanesh et al. (31)	L	H	L	U	L	Some concerns

L; low risk of bias; H, high risk of bias; U, unclear risk of bias; Overall Low Risk < 2 unclear risk of bias and no high risk of bias; Overall Some concerns = 2 unclear risk of bias and no high risk of bias; Overall High Risk > 2 unclear risk of bias or more than one high risk of bias.

### 3.4 Meta-analysis

#### 3.4.1 Effect of okra consumption on lipid profile

##### 3.4.1.1 Effect of okra consumption on serum TG levels

**Figure 2:**
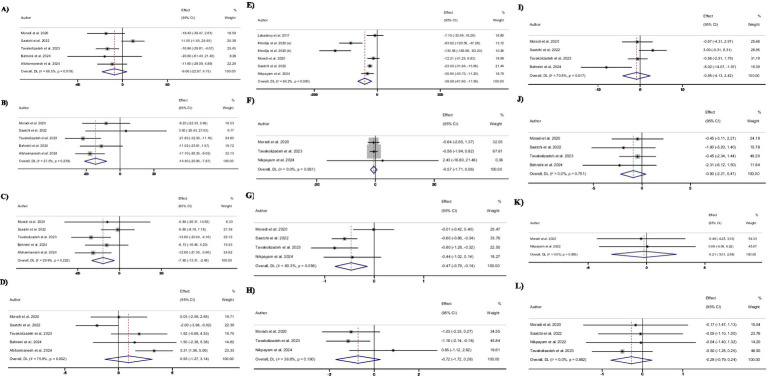
Forest plot detailing weighted mean difference and 95% confidence intervals (CIs) for the effect of Okra intake on **(A)** TG (mg/dL); **(B)** TC (mg/dL); **(C)** LDL (mg/dL); **(D)** HDL (mg/dL); **(E)** FBG (mg/dL); **(F)** insulin (uIU/mL); **(G)** HbA1c (%); **(H)** HOMA-IR; **(I)** SBP (mmHg); **(J)** DBP (mmHg); **(K)** Body weight (kg); and **(L)** BMI (kg/m^2^).

Pooling 5 included effect sizes (12, 28, 30–32), showed that Okra intake had no significant impact on TG levels compared to control groups (WMD: −9.1 mg/dL; (95% CI: −22.9 to 4.8); *p* = 0.19; Figure 2A). Also, a significant heterogeneity was detected among the included trials (*I*^2^ = 66.5%, *p* = 0.01). Furthermore, Subgroup analysis demonstrated that Okra intake led to a significant decrease in TG levels in the studies with a duration of more than 8 weeks and in studies that intervened with the dosage of Okra powder ≤3,000 mg/day (Table 3).

**Table 3:** Subgroup analyses of okra consumption on cardiometabolic risk factors in adults.

	Number of effect sizes	WMD (95%CI)	*p*- value	Heterogeneity		
				*p* heterogeneity	*I* ^2^	*p* between sub-groups
Okra consumption on serum Triglyceride (mg/dL)						
Overall effect	5	−9.1 (−22.9, 4.8)	0.19	0.01	66.5%	
Intervention type						
Extract	1	−20.0 (−61.4, 21.4)	0.34	–	–	0.59
Non-extract	4	−8.1 (−23.2, 7.0)	0.29	0.009	74.0%	
Trial duration (week)						
≤8	3	−4.9 (−23.9, 13.9)	0.60	0.02	74.4%	0.28
>8	2	−17.2 (−29.5, −4.9)	0.006	0.89	0.0%	
Powder dose (mg/day)						
≤3,000	2	−14.9 (−25.1, −4.8)	0.004	0.61	0.0%	0.42
>3,000	2	−2.3 (−31.5, 27.0)	0.87	0.01	82.3%	
Health status						
Diabetes	4	−8.8 (−27.0, 9.4)	0.34	0.009	74.1%	0.82
Prediabetes	1	−11.6 (−28.1, 4.9)	0.16	–	–	
Okra consumption on serum total Cholesterol (mg/dL)						
Overall effect	5	−14.4 (−20.9, −7.9)	<0.001	0.23	27.5%	
Intervention type						
Extract	1	−11.0 (−23.6, 1.6)	0.08	-	-	0.62
Non-extract	4	−14.8 (−23.0, −6.6)	<0.001	0.17	40.2%	
Trial duration (week)						
≤8	3	−11.3 (−21.8, −0.7)	0.03	0.18	40.9%	0.45
>8	2	−17.0 (−27.5, −6.4)	0.002	0.19	39.3%	
Powder dose (mg/day)						
≤3,000	2	−19.3 (−26.1, −12.6)	< 0.001	0.55	0.0%	0.04
>3,000	2	−5.2 (−17.4, 7.0)	0.40	0.40	0.0%	
Health status						
Diabetes	4	−12.3 (−21.4, −3.2)	0.008	0.17	39.6%	0.39
Prediabetes	1	−17.7 (−26.3, −9.1)	<0.001	–	–	
Okra consumption on serum LDL-C (mg/dL)						
Overall effect	5	−7.9 (−13.3, −2.5)	0.004	0.22	29.9%	
Intervention type						
Extract	1	−6.1 (−16.5, 4.2)	0.24	–	–	0.72
Non-extract	4	−8.4 (−15.4, −1.4)	0.01	0.13	46.3%	
Trial duration (week)						
≤8	3	−6.4 (−15.0, 2.2)	0.14	0.14	47.9%	0.51
>8	2	−10.2 (−17.5, −2.9)	0.006	0.29	8.6%	
Powder dose (mg/day)						
≤3,000	2	−13.1 (−19.5, −6.7)	<0.001	0.87	0.0%	0.02
>3,000	2	−1.5 (−8.9, 6.0)	0.69	0.65	0.0%	
Health status						
Diabetes	4	−6.4 (−12.6, −0.2)	0.04	0.24	27.2%	0.25
Prediabetes	1	−12.6 (−21.3, −3.9)	0.005	-	-	
Okra consumption on serum HDL-C (mg/dL)						
Overall effect	5	0.9 (−1.3, 3.1)	0.40	0.002	75.9%	
Intervention type						
Extract	1	1.5 (−2.4, 5.4)	0.44	–	–	0.77
Non-extract	4	0.8 (−1.7, 3.4)	0.52	0.001	81.8%	
Trial duration (week)						
≤8	3	0.5 (−2.9, 3.9)	0.78	<0.001	87.4%	0.52
>8	2	1.8 (−0.4, 4.0)	0.10	0.86	0.0%	
Powder dose (mg/day)						
≤3,000	2	2.9 (1.4, 4.3)	<0.001	0.38	0.0%	0.001
>3,000	2	−1.2 (−3.1, 0.8)	0.23	0.22	31.7%	
Health status						
Diabetes	4	0.1 (−1.8, 2.1)	0.91	0.091	53.6%	0.01
Prediabetes	1	3.3 (1.6, 5.1)	<0.001	–	–	
Okra consumption on serum FBG (mg/dL)						
Overall effect	6	−39.6 (−61.6, −17.6)	<0.001	< 0.001	84.2%	
Intervention type						
Extract	1	−31.0 (−50.7, −11.2)	0.002	-	-	0.002
Non-extract	3	−19.8 (−28.3, −11.3)	<0.001	0.33	9.3%	
Fruit	2	−105.6 (−153.8, −57.3)	<0.001	0.11	59.0%	
Trial duration (week)						
≤8	5	−43.7 (−72.0, −15.4)	0.002	< 0.001	87.3%	0.46
>8	1	−31.0 (−50.7, −11.2)	0.002	–	–	
Powder dose (mg/day)						
≤3,000	1	−7.1 (−33.5, 19.3)	0.59	–	–	0.32
>3,000	2	−21.2 (−30.1, −12.3)	<0.001	0.28	11.4%	
Baseline BMI (kg/m^2^)						
Normal (18.5–24.9)	3	−73.8 (−145.6, −1.9)	0.04	< 0.001	92.6%	0.17
Overweight (25–29.9)	1	−7.1 (−33.5, 19.3)	0.59	–	–	
Obese (>30)	2	−24.7 (−32.4, −16.9)	<0.001	0.49	0.0%	
Okra consumption on serum insulin (μU/ml)						
Overall effect	3	−0.6 (−1.7, 0.6)	0.32	0.95	0.0%	
Okra consumption on HbA1c (%)						
Overall effect	4	−0.5 (−0.8, −0.1)	0.005	0.05	60.3%	
Intervention type						
Extract	1	−0.4 (−1.0, 0.1)	0.13	–	–	0.93
Non-extract	3	−0.5 (−0.9, −0.1)	0.02	0.02	73.4%	
Trial duration (week)						
≤8	2	−0.3 (−0.9, 0.3)	0.26	0.01	82.3%	0.34
>8	2	−0.7 (−1.0, −0.3)	<0.001	0.34	0.0%	
Powder dose (mg/day)						
≤3,000	1	−0.8 (−1.3, −0.3)	0.001	–	–	0.21
>3,000	2	−0.3 (−0.9, 0.3)	0.26	0.01	82.3%	
Okra consumption on HOMA-IR						
Overall effect	3	−0.7 (−1.7, 0.3)	0.15	0.19	39.8%	
Okra consumption on SBP (mmHg)						
Overall effect	4	−0.9 (−4.1, 2.4)	0.61	0.01	70.5%	
Okra consumption on DBP (mmHg)						
Overall effect	4	−0.9 (−2.2, 0.4)	0.17	0.75	0.0%	
Okra consumption on body weight (kg)						
Overall effect	2	−0.2 (−3.0, 2.6)	0.88	0.85	0.0%	
Okra consumption on BMI (kg/m^2^)						
Overall effect	4	−0.3 (−0.8, 0.2)	0.29	0.88	0.0%	

WMD, weighted mean differences; CI, confidence interval; LDL-C, low-density lipoprotein-cholesterol; HDL-C, high-density lipoprotein cholesterol; FBG, fasting blood glucose; HbA1c, hemoglobin A1c; HOMA-IR, Homeostatic Model Assessment for Insulin Resistance; SBP, systolic blood pressure; DBP, diastolic blood pressure; BMI, body mass index.

##### 3.4.1.2 Effect of okra consumption on serum total TC levels

Combining 5 effect sizes (12, 28, 30–32), revealed that Okra consumption led to a significant decrease in total TC levels compared to control groups (WMD: −14.4 mg/dL; (95% CI: −20.9 to −7.9); *p* < 0.001; Figure 2B). Also, no significant heterogeneity was observed among the included studies (*I*^2^ = 27.5%, *p* = 0.23). Subgroup analysis showed that receiving Okra extract or Okra powder with a dosage of higher than 3,000 mg/day had no significant effect on Total TC levels.

##### 3.4.1.3 Effect of okra consumption on serum LDL-C levels

Performing meta-analyzing on the 5 included effect sizes (12, 28, 30–32), showed a significant decrease in LDL-C levels in groups that received Okra compared to the control groups (WMD: −7.9 mg/dL; (95% CI: −13.3 to −2.5); *p* = 0.004; Figure 2C). Also, no significant changes in LDL-c levels were reported by subgroup analysis in the trials with a duration of less than 8 weeks or intervention with Okra extract (*I*^2^ = 29.9%, *p* = 0.22). Furthermore, subgroup analysis demonstrated that receiving Okra powder with a dosage of higher than 3,000 mg/day led to no significant effect on LDL-C levels.

##### 3.4.1.4 Effect of okra consumption on serum HDL-C levels

Overall effect sizes estimated by combining 5 included effect sizes (12, 28, 30–32), showed that Okra intake had no significant impact on HDL-C levels compared to control groups (WMD: 0.9 mg/dL; (95% CI: −1.3 to 3.1); *p* = 0.40; Figure 2D). Furthermore, a significant heterogeneity was observed among the included trials (I^2^ = 75.9%, *p* = 0.002). Subgroup analysis reported that receiving Okra powder with a dosage of ≤3,000 mg/day led to a significant increase in HDL-C levels compared to control groups. Also, subgroup analysis revealed that Okra consumption significantly increased the HDL-C levels in individuals with prediabetes.

#### 3.4.2 Effect of okra consumption on glycemic control markers

##### 3.4.2.1 Effect of okra consumption on FBG levels

**Figure 3:**
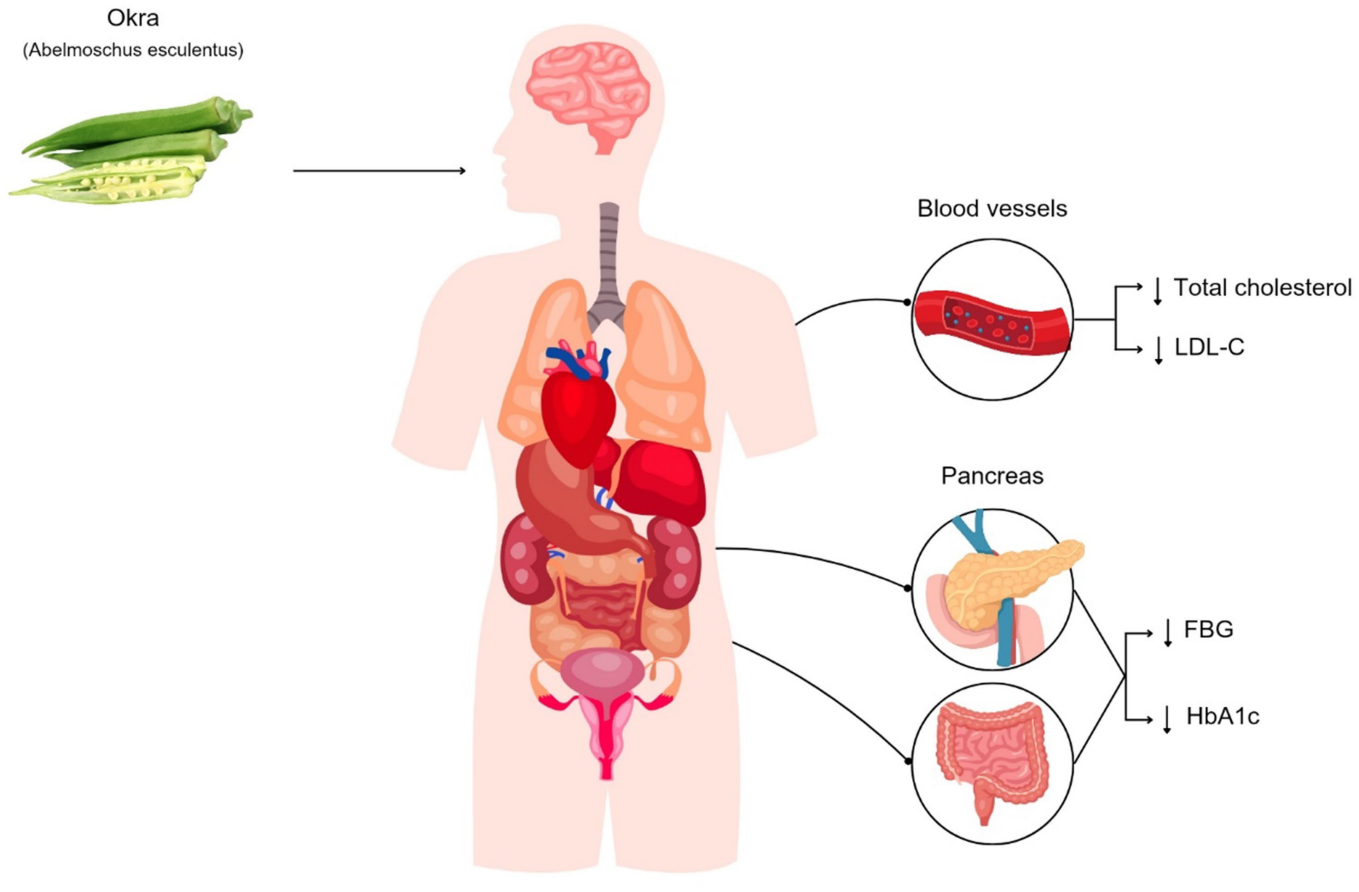
Okra consumption can reduce fasting blood glucose (FBG), hemoglobin A1c (HbA1c), low-density lipoprotein cholesterol (LDL-C), and total cholesterol in patients with diabetes.

Pooling 6 effect sizes (12, 26–28, 33), demonstrated that Okra consumption led to a significant decrease in FBG levels compared to control groups (WMD: −39.6 mg/dL; (95% CI: −61.6 to −17.6); *p* < 0.001; Figure 2E). However, significant heterogeneity was detected among the pooled trials (*I*^2^ = 84.2%, p < 0.001). Subgroup analysis showed that receiving Okra in individuals with overweight or Okra powder with a dosage of ≤3,000 mg/day had no significant impact on serum FBG levels (Figure 3).

##### 3.4.2.2 Effect of okra consumption on serum insulin levels

Combining 3 effect sizes (28, 30, 33), showed that Okra intake compared to control groups, had no significant effect on serum insulin levels (WMD: −0.6 μU/ml; (95% CI: −1.7 to 0.6); *p* = 0.32; Figure 2F). Also, there was significant heterogeneity between the included studies (*I*^2^ = 0.0%, *p* = 0.95).

##### 3.4.2.3 Effect of okra consumption on HbA1c

Combined effect sizes from 4 included studies (12, 28, 30, 33), demonstrated a significant decrease in HbA1c levels followed by Okra consumption compared to control groups (WMD: −0.5%; (95% CI: −0.8 to −0.1); *p* = 0.005; Figure 2G). In addition, no significant heterogeneity was observed among the included trials (I^2^ = 60.3%, *p* = 0.05). Subgroup analysis showed non-significant changes in HbA1c levels in the studies with a duration of ≤8 weeks or trials that intervened with Okra extract, or Okra powder with a dosage of >3,000 mg/day.

##### 3.4.2.4 Effect of okra consumption on HOMA-IR

Combining 3 effect sizes (28, 30, 33), showed that Okra intake had no significant impact on HOMA-IR levels compared to control groups (WMD: −0.7; (95% CI: −1.7 to 0.3); *p* = 0.15; Figure 2H). Also, there was no significant heterogeneity among the included trials (*I*^2^ = 39.8%, *p* = 0.19).

#### 3.4.3 Effect of okra consumption on blood pressure

##### 3.4.3.1 Effect of okra consumption on SBP

Meta-analyzing of 4 effect sizes (12, 28, 30, 32), demonstrated that Okra consumption compared to control groups, led to a non-significant changes in SBP levels compared to control groups (WMD: −0.9; (95% CI: −4.1 to 2.4); *p* = 0.61). Furthermore, significant heterogeneity was detected among the pooled studies (*I*^2^ = 70.5%, *p* = 0.01).

##### 3.4.3.2 Effect of okra consumption on DBP

Pooling 4 effect sizes (12, 28, 30, 32), showed that Okra intake had no significant impact on DBP levels compared to control groups (WMD: −0.9; (95% CI: −2.2 to 0.4); *p* = 0.17). Also, there was no significant heterogeneity among the included trials (*I*^2^ = 0.0%, *p* = 0.75).

#### 3.4.4 Effect of okra consumption on obesity indices

##### 3.4.4.1 Effect of okra consumption on body weight

Combined effect sizes that staminated by combining 2 effect sizes (28, 29), showed no significant changes in body weight followed by Okra consumption compared to control groups (WMD: −0.2; (95% CI: −3.0 to 2.6); *p* = 0.88). In addition, no significant heterogeneity was observed between the included trials (*I*^2^ = 0.0%, *p* = 0.85).

##### 3.4.4.2 Effect of okra consumption on BMI

Combining 4 effect sizes (12, 28–30), demonstrated that Okra intake had no significant impact on BMI levels compared to control groups (WMD: −0.3; (95% CI: −0.8 to 0.2); *p* = 0.29). Also, no significant heterogeneity was detected between the included trials (*I*^2^ = 0.0%, p = 0.88).

### 3.5 Sensitivity analysis

Sensitivity analysis showed that the pooled effect of Okra intake on serum TG levels after excluding the study of Saatchi et al. [WMD: −15.8 mg/dL (95%CI: −24.7, −6.9)] (12), changes significantly. Also reported that the overall size effect of Okra consumption on serum HDL-C levels after removing the trial of Saatchi et al. [WMD: 2.0 mg/dL (95%CI: 0.4, 3.5)] (12), and the overall effect size of changes in HOMA-IR levels after omitting the trial of Nikpayam et al. [WMD: -1.1 (95%CI: −1.9, −0.3)] (33), changes significantly. Furthermore, removing the effect sizes of Tavakolizadeh et al. [WMD: -0.4 (95%CI: −0.8, 0.0)] (30), or Saatchi et al. [WMD: −0.4% (95%CI: −0.9, 0.1)] (12) significantly changed the pooled effect size for HbA1c levels.

### 3.6 Publication bias

Egger’s test and visual interpretation of funnel plots did not show any significant publication bias among the pooled studies to investigate the effect of Okra on TG (P _Egger_ = 0.54), TC (P _Egger_ = 0.06), LDL-C (P _Egger_ = 0.91), HDL-C (P _Egger_ = 0.86), FBG (P _Egger_ = 0.20), Insulin (P _Egger_ = 0.30), HbA1c (P _Egger_ = 0.77), HOMA-IR (P _Egger_ = 0.22), SBP (P _Egger_ = 0.45), DBP (P _Egger_ = 0.10), and BMI (P _Egger_ = 0.13) (Supplementary Figure S1).

### 3.7 GRADE analysis

The certainty assessment of the evidence included in this review showed that the evidence of the effect of Okra on serum total cholesterol and LDL-C levels was of high quality. Also, the quality of evidence for Insulin, HbA1c, HOMA-IR, DBP, Body weight, and BMI was identified as moderate. Furthermore, the quality of evidence investigating the effect of Okra consumption on serum Triglyceride and SBP levels due to serious limitations in Inconsistency and Imprecision, and for FBG due to very serious limitations in Inconsistency was downgraded to low. Also, the quality of evidence for serum HDL-C was downgraded to very low due to the presence of a serious limitation in Imprecision and a very serious limitation of Inconsistency. The GRADE profile of certainty assessment of included evidence is presented in Table 4.

**Table 4:** Grade profile of okra consumption for cardiometabolic risk factors in adults.

Outcomes	Risk of bias	Inconsistency	Indirectness	Imprecision	Publication bias	Quality of evidence
Triglyceride	No serious limitations	Serious limitations^1^	No serious limitations	Serious limitations ^3^	No serious limitations	⊕ ⊕ ◯◯ Low
Total Cholesterol	No serious limitations	No serious limitations	No serious limitations	No serious limitations	No serious limitations	⊕ ⊕ ⊕ ⊕ High
LDL-C	No serious limitations	No serious limitations	No serious limitations	No serious limitations	No serious limitations	⊕ ⊕ ⊕ ⊕ High
HDL-C	No serious limitations	Very serious limitations^2^	No serious limitations	Serious limitations	No serious limitations	⊕◯◯◯ Very low
FBG	No serious limitations	Very serious limitations	No serious limitations	No serious limitations	No serious limitations	⊕ ⊕ ◯◯ Low
Insulin	No serious limitations	No serious limitations	No serious limitations	Serious limitations	No serious limitations	⊕ ⊕ ⊕◯ Moderate
HbA1c	No serious limitations	Serious limitations	No serious limitations	No serious limitations	No serious limitations	⊕ ⊕ ⊕◯ Moderate
HOMA-IR	No serious limitations	No serious limitations	No serious limitations	Serious limitations	No serious limitations	⊕ ⊕ ⊕◯ Moderate
SBP	No serious limitations	Serious limitations	No serious limitations	Serious limitations	No serious limitations	⊕ ⊕ ◯◯ Low
DBP	No serious limitations	No serious limitations	No serious limitations	Serious limitations	No serious limitations	⊕ ⊕ ⊕◯ Moderate
Body weight	No serious limitations	No serious limitations	No serious limitations	Serious limitations	No serious limitations	⊕ ⊕ ⊕◯ Moderate
BMI	No serious limitations	No serious limitations	No serious limitations	Serious limitations	No serious limitations	⊕ ⊕ ⊕◯ Moderate

1. There is high heterogeneity (*I*^2^ > 40%). 2. There is high heterogeneity (*I*^2^ > 75%). 3. There is no significant effect of Okra consumption.

## 4 Discussion

The present systematic review and meta-analysis study showed that okra decreased TC, LDL, FBG, and HbA1c levels in the intervention group compared to the control group. Also, the consumption of okra in powder form compared to the extract caused a significant decrease in TC, LDL, and HbA1c, while the consumption of its fruit caused a greater decrease in FBG compared to the other two forms. The intervention duration ˃ 8 weeks led to a significant reduction of TG, and HbA1c, and the reduction of TC with the intervention > 8 weeks was greater than the intervention ≤8 weeks, while this issue was the opposite for FBG. A dose ≤3,000 mg/day caused a significant decrease in TG, TC, LDL, HbA1c, and a significant increase in HDL, while FBG had a significant decrease in a dose > 3,000 mg/day. The reduction of TC and LDL was higher in subjects with diabetes compared to subjects with diabetes. In subjects with pre-diabetes, the increase in HDL was statistically significant. FBG reduction was significant in obese subjects. There were no significant differences in the parameters of insulin level, HOMA-IR, systolic and diastolic blood pressure, weight, and BMI.

Mokgalaboni et al.’s systematic review and meta-analysis study on 8 RCTs in patients with diabetes and prediabetes showed that okra, similar to the results of the present meta-analysis, reduced FBG, while it had no effect on HbA1c (14). While Mokgalaboni’s study focused specifically on glycemic control in prediabetes and type 2 diabetes (T2D), our study has a broader scope, examining a range of cardiometabolic risk factors, including lipid profiles (total cholesterol, LDL, HDL, triglycerides) as well as blood pressure, insulin resistance (HOMA-IR), and body weight in individuals with prediabetes and diabetes. The number of studies reviewed in Mokgalaboni’s meta-analysis was less than the present review and the subgroup based on the dose and duration of the intervention and the participants and the intervention type was not done.

In a recent meta-analysis, the positive effects of okra on lipid profiles were demonstrated. This study included not only diabetic and prediabetic patients but also obese individuals, revealing reductions in LDL and total cholesterol, as well as decreases in triglycerides and increases in HDL (34). In the systematic animal review study by Sereno et al., which examined 11 studies, the effect of okra on glycemic control markers (glycated hemoglobin, HOMA-IR, glucose tolerance test, and blood glucose) and cholesterol was shown in animals with diabetes (35). The systematic review study by Nikpayam et al., who reviewed 54 human and animal studies, also found improvement effects in glycemic control and lipid profile indicators (36). Other differences between the previous studies and the present study include the investigation of diastolic and systolic blood pressure parameters, as well as weight and BMI, which we examined in this study.

The mechanisms by which okra exerts improvement effects on cardiometabolic factors include:

Okra reduces blood glucose levels and HbA1c through a combination of its high fiber content, mucilage, antioxidant properties, inhibition of carbohydrate-digesting enzymes including *α*-amylase and α-glucosidase, and improvement of insulin sensitivity (14, 30, 37, 38). One suggested way okra helps lower FBG levels involves promoting liver glycogen formation and regenerating pancreatic islets. This results in enhanced insulin release and a slower absorption of glucose in the intestines (39). Additional mechanisms thought to contribute to okra’s anti-hyperglycemic effect involve enhancing glucose regulation and repairing *β*-cell dysfunction via a pathway dependent on peroxisome proliferator-activated receptors (PPAR) (40). The abundant phenols in okra enhance insulin sensitivity and pancreatic β-cell performance by decreasing reactive oxygen species (ROS) (41). Notably, okra is rich in antioxidants, including flavonoids, quercetin, polyphenols, and vitamins A and C (42). These combined effects help slow down the absorption of carbohydrates, improve glucose metabolism, and ultimately lead to better glycemic control.

Okra can improve lipid profiles through its high content of soluble fiber and mucilage, which bind to bile acids and cholesterol, enhancing their excretion (43). Non-esterified fatty acids (NEFAs) enhance the production of lipogenesis-related molecules, including sterol regulatory element-binding protein 1c (SREBP1c) and fatty acid synthase (FAS) (44). Additionally, they suppress the production of lipolysis-related molecules such as carnitine palmitoyltransferase 1A (CPT1A) and hormone-sensitive lipase (HSL) (44). Okra reduces the level of NEFAs (34). Some studies propose that the lipid-lowering effect of *Abelmoschus esculentus* is linked to an increase in lipogenesis by suppressing the activity of cholesterol-7-*α*-hydroxylase (CYP7A1) and fatty acid synthase (FAS) (45). Its antioxidant properties, due to polyphenols and flavonoids, reduce oxidative stress and inflammation, which helps prevent LDL cholesterol oxidation (41). Additionally, saponins in okra reduce cholesterol absorption, while its effects on lipid metabolism genes and gut microbiota further contribute to improved lipid profiles (30, 36, 38). Okra appears to enhance HDL by boosting the activity of LDL receptors (LDLR), facilitating the removal of LDL (43). Furthermore, the abundant polyphenols and flavonoids in okra help maintain elevated HDL levels by preventing the oxidation of LDL (46, 47). These combined mechanisms help lower total cholesterol, LDL cholesterol, and triglycerides while potentially increasing HDL cholesterol, thus supporting cardiovascular health.

A dose of okra ≤3,000 mg per day may be more effective in improving blood lipids due to optimal absorption and utilization, avoidance of gastrointestinal side effects, balanced nutrient intake, potential hormetic effects, efficient metabolic processing, better bioavailability, and enhanced long-term adherence. These factors collectively ensure that the beneficial compounds in okra exert their lipid-lowering effects more effectively without overwhelming the body’s systems (30, 48).

While okra supplements can offer health benefits, they may also cause side effects such as gastrointestinal issues, increased risk of kidney stones, potential blood glucose control issues, allergic reactions, nutrient absorption interference, drug interactions, and digestive enzyme interference. It is advisable for individuals to consult with a healthcare provider before starting okra supplements, especially those with pre-existing health conditions or those taking other medications. Moderation and adherence to recommended dosages can help minimize these potential side effects (48, 49).

### 4.1 Clinical insight

Among the examined parameters, the change of FBG level is higher than the minimal clinically important difference (MCID) which is 5–10 mg/dL (50), which means that okra can be effective as a clinically effective food supplement in reducing FBG.

### 4.2 Strength and limitation

The present study comprehensively investigated the effect of okra on cardiometabolic factors. In addition, subgroup analysis and GRADE analysis are considered to be one of its strengths. All included RCTs had a low bias. One of the limitations of the present study is the small number of RCTs so subgroup analysis was not possible for some variables. Also, the sensitivity analysis for some variables indicated bias and the GRADE analysis for all variables except for TC indicated moderate to very low certainty. Other limitations include high heterogeneity, different forms of okra used, no indication of which part of okra was used (leaves, stem, seed, etc), different doses and duration of intervention, and different conditions (prediabetes, type 2 diabetes and diabetic nephropathy). The evidence presented all are from Asia (thus, the results may be specific to the population, making it difficult to generalize them to other continents, especially considering differences in diet, lifestyle, and genetics between populations), these could be a very great limitation as the results might not be the same in Europe and African countries. Also, we acknowledge that the included okra treatments vary significantly in their effects due to differences in form (whole vs. powder), processing methods, and nutrient composition. This variability may influence gastric filling, taste, and satiety, which we recognize as a limitation in our study. Future research should aim to differentiate these effects to provide a clearer understanding of the impact of various okra preparations on health outcomes.

## 5 Conclusion

This systematic review and meta-analysis found that okra consumption significantly reduced TC, LDL, FBG and HbA1c levels. Powdered okra and longer interventions were more effective, with doses of 3,000 mg or less also increasing HDL. Effects were more pronounced in diabetics for TC and LDL, pre-diabetics for HDL, and obese subjects for FBG, with no significant changes in insulin, blood pressure, weight, or BMI. However, in order to determine the optimum dose and duration, more and better quality RCTs are needed.

## Data Availability

The original contributions presented in the study are included in the article/Supplementary material, further inquiries can be directed to the corresponding author.

## References

[1] Putri S Ciminata G Lewsey J Jani B McMeekin N Geue C. The conceptualisation of cardiometabolic disease policy model in the UK. BMC Health Serv Res. (2024) 24:1060. doi: 10.1186/s12913-024-11559-y, PMID: 39272116 PMC11396645

[2] Ndisang JF Rastogi S. Cardiometabolic diseases and related complications: current status and future perspective. Biomed Res Int. (2013) 2013:467682. doi: 10.1155/2013/467682 24224165 PMC3809929

[3] Sohouli MH Lari A Fatahi S Shidfar F Găman M-A Guimaraes NS . Impact of soy milk consumption on cardiometabolic risk factors: a systematic review and meta-analysis of randomized controlled trials. J Funct Foods. (2021) 83:104499. doi: 10.1016/j.jff.2021.104499

[4] Buitrago-Lopez A Sanderson J Johnson L Warnakula S Wood A Di Angelantonio E . Chocolate consumption and cardiometabolic disorders: systematic review and meta-analysis. BMJ. (2011) 343:d4488. doi: 10.1136/bmj.d4488, PMID: 21875885 PMC3163382

[5] Asbaghi O Ashtary-Larky D Mousa A Rezaei Kelishadi M Moosavian SP. The effects of soy products on cardiovascular risk factors in patients with type 2 diabetes: a systematic review and Meta-analysis of clinical trials. Adv Nutr (Bethesda, Md). (2022) 13:455–73. doi: 10.1093/advances/nmab121, PMID: 34591084 PMC8970819

[6] Elkhalifa AEO Alshammari E Adnan M Alcantara JC Awadelkareem AM Eltoum NE . Okra (*Abelmoschus esculentus*) as a potential dietary medicine with nutraceutical importance for sustainable health applications. Molecules. (2021) 26:696. doi: 10.3390/molecules26030696 33525745 PMC7865958

[7] Gomes MFP de Moura EOC Cardoso NM da Silva GA Dos Santos ACC de Souza FS . Supplementation with okra combined or not with exercise training is able to protect the heart of animals with metabolic syndrome. Sci Rep. (2023) 13:1468. doi: 10.1038/s41598-023-28072-7, PMID: 36702820 PMC9879946

[8] Esmaeilzadeh D Razavi BM Hosseinzadeh H. Effect of *Abelmoschus esculentus* (okra) on metabolic syndrome: a review. Phytother Res. (2020) 34:2192–202. doi: 10.1002/ptr.6679, PMID: 32222004

[9] Atawodi SE Atawodi JC Idakwo GA Pfundstein B Haubner R Wurtele G . Polyphenol composition and antioxidant potential of *Hibiscus esculentus* L. fruit cultivated in Nigeria. J Med Food. (2009) 12:1316–20. doi: 10.1089/jmf.2008.0211, PMID: 20041787

[10] Malek Mahdavi A Javadivala Z Ahmadian E. Effects of okra (*Abelmoschus esculentus* L) on inflammatory mediators: a systematic review of preclinical studies. Food Funct. (2022) 13:3159–69. doi: 10.1039/D1FO03915F, PMID: 35244638

[11] Kuruwita Arachchige S Uluwaduge DI Premakumara S Wijayabandara J. Cardio protective activity of *Abelmoschus esculentus* (okra). Int J Food Sci Nutr. (2018) 3:39–43.

[12] Saatchi A Aghamohammadzadeh N Beheshtirouy S Javadzadeh Y Afshar FH Ghaffary S. Anti-hyperglycemic effect of Abelmoschus culentesus (okra) on patients with diabetes type 2: a randomized clinical trial. Phytother Res. (2022) 36:1644–51. doi: 10.1002/ptr.7341, PMID: 35434945

[13] Zhang T Xiang J Zheng G Yan R Min X. Preliminary characterization and anti-hyperglycemic activity of a pectic polysaccharide from okra (*Abelmoschus esculentus* (L.) Moench). J Funct Foods. (2018) 41:19–24. doi: 10.1016/j.jff.2017.12.028

[14] Mokgalaboni K Lebelo SL Modjadji P Ghaffary S. Okra ameliorates hyperglycaemia in pre-diabetic and type 2 diabetic patients: a systematic review and meta-analysis of the clinical evidence. Front Pharmacol. (2023) 14:1132650. doi: 10.3389/fphar.2023.1132650, PMID: 37077817 PMC10107009

[15] Page MJ McKenzie JE Bossuyt PM Boutron I Hoffmann TC Mulrow CD . The PRISMA 2020 statement: an updated guideline for reporting systematic reviews. Int J Surg. (2021) 88:105906. doi: 10.1016/j.ijsu.2021.105906, PMID: 33789826

[16] Cumpston M Li T Page MJ Chandler J Welch VA Higgins JP . Updated guidance for trusted systematic reviews: a new edition of the Cochrane handbook for systematic reviews of interventions. Cochrane Database Syst Rev. (2019) 2019:ED000142. doi: 10.1002/14651858.ED000142 PMC10284251 31643080

[17] DerSimonian R Laird N. Meta-analysis in clinical trials. Control Clin Trials. (1986) 7:177–88. doi: 10.1016/0197-2456(86)90046-2 3802833

[18] Jazinaki MS Bahari H Rashidmayvan M Arabi SM Rahnama I Malekahmadi M. The effects of raspberry consumption on lipid profile and blood pressure in adults: a systematic review and meta-analysis. Food Sci Nutr. (2024) 12:2259–78. doi: 10.1002/fsn3.3940, PMID: 38628181 PMC11016397

[19] Borenstein M Hedges LV Higgins JP Rothstein HR. Introduction to meta-analysis. New York: John Wiley and Sons (2021).

[20] Hozo SP Djulbegovic B Hozo I. Estimating the mean and variance from the median, range, and the size of a sample. BMC Med Res Methodol. (2005) 5:1–10. doi: 10.1186/1471-2288-5-13 15840177 PMC1097734

[21] Wan X Wang W Liu J Tong T. Estimating the sample mean and standard deviation from the sample size, median, range and/or interquartile range. BMC Med Res Methodol. (2014) 14:135. doi: 10.1186/1471-2288-14-135 25524443 PMC4383202

[22] Higgins JP Thompson SG Deeks JJ Altman DG. Measuring inconsistency in meta-analyses. BMJ. (2003) 327:557–60. doi: 10.1136/bmj.327.7414.557, PMID: 12958120 PMC192859

[23] Bahari H Pourreza S Goudarzi K Mirmohammadali SN Asbaghi O Kolbadi KSH . The effects of pomegranate consumption on obesity indices in adults: a systematic review and meta-analysis. Food Sci Nutr. (2024) 12:641–60. doi: 10.1002/fsn3.3739, PMID: 38370057 PMC10867489

[24] Higgins JP Thompson SG. Quantifying heterogeneity in a meta-analysis. Stat Med. (2002) 21:1539–58. doi: 10.1002/sim.1186, PMID: 12111919

[25] Guyatt GH Oxman AD Vist GE Kunz R Falck-Ytter Y Alonso-Coello P . GRADE: an emerging consensus on rating quality of evidence and strength of recommendations. BMJ. (2008) 336:924–6. doi: 10.1136/bmj.39489.470347.AD, PMID: 18436948 PMC2335261

[26] Labadnoy WA Lacas C Frances ML Lauren DB Alberto LJ Lim KM . The efficacy of okra (*Abelmoschus esculentus*) in decreasing blood sugar levels among patients with impaired fasting glucose in Antipolo City. UERM Health Sci J. (2017) 1:6.

[27] Khodija U Wiboworini B Kartikasari L. Comparing the effect of steamed and boiled okra (*Abelmoschus esculentus*) on fasting blood glucose among type 2 diabetes mellitus patients with hypercholesterolemia. Int J Nutr Sci. (2020) 5:65–71.

[28] Moradi A Tarrahi MJ Ghasempour S Shafiepour M Clark CC Safavi SM. The effect of okra (*Abelmoschus esculentus*) on lipid profiles and glycemic indices in type 2 diabetic adults: randomized double blinded trials. Phytother Res. (2020) 34:3325–32. doi: 10.1002/ptr.6782, PMID: 32706159

[29] Nikpayam O Safaei E Bahreyni N Sadra V Saghafi-Asl M Fakhr L. The effect of *Abelmoschus esculentus* L.(okra) extract supplementation on dietary intake, appetite, anthropometric measures, and body composition in patients with diabetic nephropathy. Health promotion. Perspectives. (2022) 12:169–77. doi: 10.34172/hpp.2022.21 PMC9508394 36276416

[30] Tavakolizadeh M Peyrovi S Ghasemi-Moghaddam H Bahadori A Mohkami Z Sotoudeh M . Clinical efficacy and safety of okra (*Abelmoschus esculentus* (L.) Moench) in type 2 diabetic patients: a randomized, double-blind, placebo-controlled, clinical trial. Acta Diabetol. (2023) 60:1685–95. doi: 10.1007/s00592-023-02149-1, PMID: 37507536

[31] Afsharmanesh MR Mansourian AR Saghaeian Jazi M Ghaffary S Eshghinia S Behnampour N . Okra (*Abelmoschus esculentus*) intake improves lipid profile and liver transaminases in pre-diabetic adults: a randomized double-blinded trial. Jundishapur J Nat Pharm Prod. (2024) 19:74. doi: 10.5812/jjnpp-143074

[32] Bahreini N Saghafi-Asl M Nikpayam O Safaei E Sadra V Fakhr L . Effects of dried okra extract on lipid profile, renal function and some RAGE-related inflammatory genes expression in patients with diabetic nephropathy: a randomized controlled trial. Complement Ther Med. (2024) 81:103027. doi: 10.1016/j.ctim.2024.103027 38336011

[33] Nikpayam O Saghafi-Asl M Safaei E Bahreyni N Sadra V Asgharian P. The effect of *Abelmoschus esculentus* L.(okra) extract supplementation on glycaemic control, inflammation, kidney function and expression of PPAR-α, PPAR-γ, TGF-β and Nrf-2 genes in patients with diabetic nephropathy: a triple-blind, randomised, placebo-controlled trial. Br J Nutr. (2024) 131:648–57. doi: 10.1017/S0007114523002180, PMID: 37840235

[34] Mokgalaboni K Phoswa WN Mokgalabone TT Dlamini S Ndhlala AR Modjadji P . Effect of *Abelmoschus esculentus* L. (okra) on dyslipidemia: systematic review and Meta-analysis of clinical studies. Int J Mol Sci. (2024) 25:922. doi: 10.3390/ijms252010922 39456704 PMC11507881

[35] Sereno AB Dayane Pinto C Antunes Andrade F Bertolazo A da Silva M Carvalho Garcia A . Effects of okra (*Abelmoschus esculentus* (L.) Moench) on glycemic markers in animal models of diabetes: a systematic review. J Ethnopharmacol. (2022) 298:115544. doi: 10.1016/j.jep.2022.115544, PMID: 35963420

[36] Nikpayam O Safaei E Bahreini N Saghafi-Asl M. The effects of okra (*Abelmoschus esculentus* L.) products on glycemic control and lipid profile: a comprehensive systematic review. J Funct Foods. (2021) 87:104795. doi: 10.1016/j.jff.2021.104795

[37] Amadi JA Amadi PU Njoku UC. Okra modulates regulatory enzymes and metabolites of glucose-utilizing pathways in diabetic rats. J Am Coll Nutr. (2021) 40:689–98. doi: 10.1080/07315724.2020.1815249, PMID: 33031022

[38] Fan S Zhang Y Sun Q Yu L Li M Zheng B . Extract of okra lowers blood glucose and serum lipids in high-fat diet-induced obese C57BL/6 mice. J Nutr Biochem. (2014) 25:702–9. doi: 10.1016/j.jnutbio.2014.02.010, PMID: 24746837

[39] Abbas A Muhammad I AbdulRahman M Bilbis L Saidu Y Onu A. Possible antidiabetic mechanism of action of ex-Maradi okra fruit variety (*Abelmoscus esculentus*) on alloxan induced diabetic rats. Niger J Basic Appl Sci. (2017) 25:101–13. doi: 10.4314/njbas.v25i2.11

[40] Majd NE Tabandeh MR Shahriari A Soleimani Z. Okra (*Abelmoscus esculentus*) improved islets structure, and down-regulated PPARs gene expression in pancreas of high-fat diet and streptozotocin-induced diabetic rats. Cell J (Yakhteh). (2018) 20:31. doi: 10.22074/cellj.2018.4819 PMC5759678 29308616

[41] Patel RP Barnes S. Isoflavones and PPAR signaling: a critical target in cardiovascular, metastatic, and metabolic disease. PPAR Res. (2010) 2010:153252. doi: 10.1155/2010/153252 21461045 PMC3061262

[42] Xia F Zhong Y Li M Chang Q Liao Y Liu X . Antioxidant and anti-fatigue constituents of okra. Nutrients. (2015) 7:8846–58. doi: 10.3390/nu7105435, PMID: 26516905 PMC4632455

[43] Panighel G Ferrarese I Lupo MG Sut S Dall'Acqua S Ferri N. Investigating the in vitro mode of action of okra (*Abelmoschus esculentus*) as hypocholesterolemic, anti-inflammatory, and antioxidant food. Food Chem. (2022) 5:100126. doi: 10.1016/j.fochms.2022.100126 PMC9352527 35937040

[44] Fan X Xu J Hu Y Wang K Zhao Y Cai J . Effect of high NEFA concentration on lipid metabolism disorders in hepatocytes based on lipidomics. Front Pharmacol. (2024) 15:296. doi: 10.3389/fphar.2024.1372296, PMID: 38482059 PMC10933074

[45] Wang H Chen G Ren D Yang S-T. Hypolipidemic activity of okra is mediated through inhibition of lipogenesis and upregulation of cholesterol degradation. Phytother Res. (2014) 28:268–73. doi: 10.1002/ptr.4998, PMID: 23606408

[46] Mohammed M Bayero A Shettima U. Levels of total phenolic and flavonoids in *Abelmoschus esculentus* L. from some irrigation areas of Kano state-Nigeria. Bayero J Pure Appl Sci. (2016) 9:121–4. doi: 10.4314/bajopas.v9i2.23

[47] Yang J Chen X Rao S Li Y Zang Y Zhu B. Identification and quantification of flavonoids in okra (*Abelmoschus esculentus* L. Moench) and antiproliferative activity in vitro of four main components identified. Meta. (2022) 12:483. doi: 10.3390/metabo12060483 PMC9228595 35736417

[48] Badrie N. Nutrient profile, bioactive components, and functional properties of okra (*Abelmoschus esculentus* (L.) Moench). Fruits Vegetables Herbs. (2016) 1:365–409. doi: 10.1016/B978-0-12-802972-5.00018-4

[49] Punukollu RS Chadalawada AK Siddabattuni K Gogineni NT. A blend of *Withania somnifera* (L.) Dunal root and *Abelmoschus esculentus* (L.) Moench fruit extracts relieves constipation and improves bowel function: a proof-of-concept clinical investigation. J Ethnopharmacol. (2024) 318:116997. doi: 10.1016/j.jep.2023.116997, PMID: 37543151

[50] Banks J Amspoker AB Vaughan EM Woodard L Naik AD. Ascertainment of minimal clinically important differences in the diabetes distress scale–17: a secondary analysis of a randomized clinical trial. JAMA Netw Open. (2023) 6:950. doi: 10.1001/jamanetworkopen.2023.42950 PMC10652154 37966840

