# Supplementary material for: The cardiometabolic benefits of okra-based treatment in prediabetes and diabetes: a systematic review and meta-analysis of randomized controlled trials
Source: Front Nutr. 2024 Dec 12;11:1454286. doi: 10.3389/fnut.2024.1454286 (PMC11670074; doi:10.3389/fnut.2024.1454286)
Supplement: Supplementary file 1 [file Table_1.DOCX]

A)

B)

C)

D)

E)

F)

G)

H)

I)

J)

K)

L)

**Figure 3.** Funnel plots for the effect of Okra intake on A) TG (mg/dL); B) TC (mg/dL); C) LDL (mg/dL); D) HDL (mg/dL); E) FBG (mg/dL); F) insulin (uIU/mL); G) HbA1c (%); H) HOMA-IR; I) SBP (mmHg); J) DBP (mmHg); K) Body weight (kg); and L) BMI (kg/m²).
